# Availability and End-to-end Reliability in Low Duty Cycle Multihop Wireless Sensor Networks

**DOI:** 10.3390/s90302088

**Published:** 2009-03-20

**Authors:** Jukka Suhonen, Timo D. Hämäläinen, Marko Hännikäinen

**Affiliations:** Tampere University of Technology / Department of Computer Systems, P.O.Box 553, FIN-33101 Tampere, Finland; E-Mails: timo.d.hamalainen@tut.fi (T.D.H.), marko.hannikainen@tut.fi (M.H.A.)

**Keywords:** Wireless sensor networks, reliability, availability, QoS

## Abstract

A wireless sensor network (WSN) is an ad-hoc technology that may even consist of thousands of nodes, which necessitates autonomic, self-organizing and multihop operations. A typical WSN node is battery powered, which makes the network lifetime the primary concern. The highest energy efficiency is achieved with low duty cycle operation, however, this alone is not enough. WSNs are deployed for different uses, each requiring acceptable Quality of Service (QoS). Due to the unique characteristics of WSNs, such as dynamic wireless multihop routing and resource constraints, the legacy QoS metrics are not feasible as such. We give a new definition to measure and implement QoS in low duty cycle WSNs, namely availability and reliability. Then, we analyze the effect of duty cycling for reaching the availability and reliability. The results are obtained by simulations with ZigBee and proprietary TUTWSN protocols. Based on the results, we also propose a data forwarding algorithm suitable for resource constrained WSNs that guarantees end-to-end reliability while adding a small overhead that is relative to the packet error rate (PER). The forwarding algorithm guarantees reliability up to 30% PER.

## 1. Introduction

A wireless sensor network (WSN) is an ad-hoc network technology that may consist of thousands of autonomic and self-organizing nodes. To achieve low cost, WSN nodes use components that impose severe resource and power constraints. A typical WSN node has only a couple of Millions of Instructions Per Second (MIPS) processing speed, tens of kilobytes program and data memories, and error prone low power radio connectivity. As a result, a WSN node combines environmental sensing, data processing, and wireless networking in constantly changing environment with extremely low energy and cost. The applications for sensor networks range from home and industrial environments to military uses.

Due to the *ad-hoc* nature of the network, WSN nodes are battery powered, which makes the network lifetime the primary concern [1]. The highest energy efficiency can be achieved with low duty cycle operation, in which a node saves energy in a low power state and is active only a fraction of time. This is the normal method in most proposals and standards in WSNs [2–4]. Although the low duty cycle operation reduces capacity and increases delays, it is deemed suitable for WSNs as traffic load is usually light and network lifetime is a key factor.

WSNs are deployed for different uses, each requiring unique Quality of Service (QoS). In legacy communication networks, QoS is commonly expressed and managed by throughput, latency, jitter, and reliability. However, due to the unique characteristics, especially dynamic wireless multihop routing and resource constraints, these are not feasible for WSNs as such. QoS varies as network load, number of nodes, topology, and operating conditions change. Problems occurring on a single link propagate via established routes to other parts of network. Still, a sensor reading may indicate a potentially life-threatening situation, thus requiring timely delivery.

**Figure 1.**
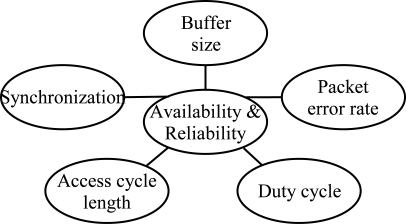
Factors affecting availability and reliability evaluated in this paper.

The scope of this paper is detailed in Figure 1. In this paper, we analyze how QoS is affected by low duty cycling, synchronization, unreliable wireless communications causing packet errors, and buffer space limitations due to the resource constraints. For energy, we assume the trade-off between performance and battery life to be a result of duty cycling. To save more energy, lower duty cycle is applied.

We have concluded two methods to measure QoS in low duty cycle WSNs. The first is availability, which expresses the probability to receive a new measurement from a node within a certain waiting period. The second is reliability, which means the probability to transfer a single measurement through the network within certain latency. Buffer space control, duty cycle control, and access cycle length adjustment are methods for managing the availability and reliability, and used in the evaluations to present factors for resulting performance. Based on the results, we propose a data forwarding algorithm suitable for resource constrained WSNs to guarantee end-to-end reliability.

For realistic results, availability and reliability are evaluated in two low power, multihop WSNs: ZigBee [5] and TUTWSN. These network technologies are not compared to each other, but represent different approaches to channel access and routing. ZigBee technology is emerging as a *de-facto* standard for WSNs, and represents a general contention-based radio link technology. TUTWSN, on the other hand, is a university proposal which represents a general contention-free link technology using Time Division Multiple Access (TDMA) without carrier sensing.

To our knowledge, this paper is the first to evaluate the effect of duty cycling to availability and reliability in WSNs, and to present such analysis on ZigBee and a TDMA-based large scale WSNs.

The rest of the paper is organized as follows. Availability and reliability in WSNs discussed Section 2, including analysis of related research. Section 3 presents the ZigBee and TUTWSN technologies. Simulation cases and results are given in Section 4. In Section 5, we propose a simple but efficient data forwarding algorithm which increases reliability and availability in both technology cases. Section 6 concludes the paper.

## 2. End-to-End QoS in WSNs

While the traditional reliability, latency, and throughput QoS parameters also apply to the WSNs, their importance differs from the legacy communication networks. In WSNs, sensing applications can tolerate high latency and low throughput. However, the reliability in WSNs is particularly significant. In the traditional computer networks, the data is routed via highly reliable wired links, while only the end links may be wireless, for example by utilizing cellular connections or Wireless LAN (WLAN). In WSNs, packets are forwarded via multiple wireless hops. On each wireless link, the packet error rates (PER) of 10%–30% are common [6, 7], which significantly decreases the end-to-end reliability. For example, assuming 30% PER and 3 transmission attempts on each link, 97% of packets are received over one hop based on a simple probability calculation. After 10 hops, the success probability is only 76%.

**Figure 2.**
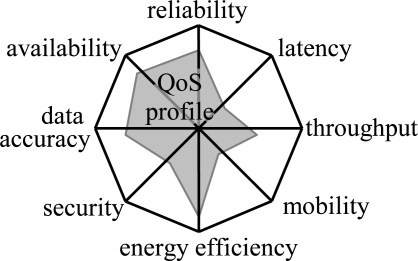
Quality of service (QoS) parameters in WSNs. The shown network QoS profile emphasizes reliability, availability, data accuracy, and energy-efficiency.

There are other aspects in QoS, which are outside the scope of this paper. However, as an introduction we can identify data accuracy, security, mobility, and energy efficiency parameters. These constitute a profile that describes the type of QoS in a network as shown in Figure 2. The data accuracy and security parameters emphasize the data centric nature of the WSNs. The data accuracy describes the accuracy of sensor measurements, whereas the security ensures that unauthorized parties do not gain access or tamper with the sensed data. The mobility is important in tracking WSNs as a node may be attached to moving objects. Due to the significance of the network lifetime, energy efficiency is considered as a QoS parameter. Usually, the other parameters have a trade-off with the energy efficiency. For example, throughput can be increased with a higher duty cycle thus reducing sleeping while increasing energy usage.

### 2.1. Low Duty Cycle Operation

**Figure 3.**
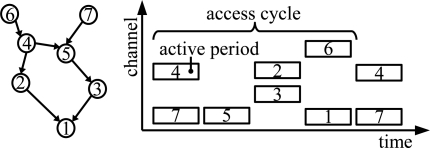
Sleep schedules in a low duty cycle network. A node forwards data during the active period of a neighbor node.

In the low duty cycle operation, a node maintains periodic sleep schedule, referred to as an access cycle, that consists of an active and idle period. The duty cycle refers to the ratio of active time to total time. A node receives data during its active period. During the idle period, a node forwards data to its neighbors or saves energy by sleeping. An example of the sleep schedules in a multihop network is presented in Figure 3.

The periodic sleeping necessitates synchronization between nodes, which is commonly realized by transmitting beacon frames at the beginning of an active period. For energy efficiency, nodes may form clusters that use a dedicated node referred to as cluster head to transmit beacons [8, 9]. A cluster head is also responsible for routing and forwarding data from its member nodes to other clusters.

The cluster heads that are within interference range may not have overlapping active periods. Otherwise, the transmissions from different clusters might collide and cause packet loss. As a result, the cluster density is limited by the number of active periods within an access cycle. To increase this number, clusters may operate on different channels. This way, clusters do not interfere each other even if their active periods overlap.

### 2.2. Availability and End-to-end Reliability

In this paper, reliability expresses the probability of a successful packet delivery from a source to the destination. As the delivery time has usually a limit after which a packet becomes useless, the reliability is evaluated together with latency. Alert and control messages are the types of WSN traffic that require high reliability and low latencies.

As the wireless connections are unreliable, it is essential to know how reliably the sensor readings can be collected. In monitoring networks, it is important to find the longest (worst) time between received updates. This cannot be calculated from the average reliability, as a network may function correctly for a long time and then experience a momentarily failure e.g. due to a link break. To express the time required to recover from errors, we use availability as a second metric. The availability is expressed in nines notation, e.g. the availability of two nines means that information has been received from a node 99% of the time.

We define the availability as a probability that data is received from a node within certain time interval *I* as

(1) $$\mathrm{availability}=\frac{\left|\{1\leq i\leq N-1:a_{i}-a_{i-1}\leq I\}\right|}{N-1},$$

where *a_i_* is the arrival time of the *i*th sample, *N* is the number of received samples. Thus, a node is considered available when sensor values are received from it over the time of observation.

**Figure 4.**
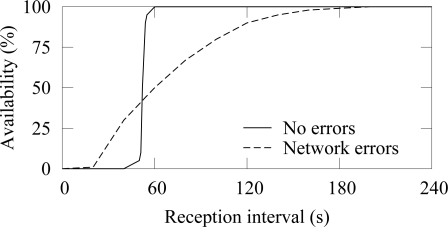
Availability metric expressing the probability that an update is received from a node within certain time interval. Packet drops and network errors decrease the availability.

An example of the availability when a node generates traffic at constant 50 s intervals is shown in Figure 4. ¿From (1) it follows that the measured availability resembles the cumulative distribution function (cdf) of the process that generates traffic at a source node. Therefore, when errors do not occur, the average reception interval is 50 s. Due to variation in end-to-end latencies, the cdf spreads slightly and the availability reaches 100% at 60 s. When packet errors occur, variation around the average reception interval increases and reaching high availability requires a large reception interval. Packet losses increase the time between receptions increases and consequently decrease the availability. In the example, 99% availability is reached only after 180 s reception interval.

In practice, the availability can be used to evaluate the applicability of the network for a certain purpose. For example, in a WSN that is targeted at intruder detection may not be unavailable for a long time or otherwise an alert can be received too late. Thus, an availability (e.g. 99.99%) must be associated with a time interval, e.g. one minute. In a measurement network the interval may be even ten minutes as the observed phenomena changes slowly.

The availability can be measured in any network by instructing nodes to send periodic keep-alive messages. The simulations in this paper use a typical measurement WSN, in which the availability is naturally calculated from the periodic updates.

### 2.3. Causes for Unreliability

In wireless networks, packet errors are common due to fading caused by environment and interference from other wireless devices. WSNs are even more prone to errors as they may operate on hostile environments. Also, as nodes are often randomly deployed, the placing and the distances between nodes can be unideal, thus causing unreliable links.

The synchronization via beacons has two major impacts on performance and reliability. First, missing a beacon prevents a node from participating in the following active period, therefore preventing a node forwarding its data. Second, several missed beacons indicate synchronization loss, which necessitates searching for neighbors with a time consuming network scan. For example, if a network uses 4 s access cycle and 10 channels, it takes 40 s to perform a complete network scan. During this time, a node cannot forward any data and generated packets must be buffered.

Packet buffering in WSNs is limited due to the memory constraints. When a buffer is full, a packet must be dropped, which reduces reliability. Low power WSN platforms often depend on the data memory supplied by the MicroController Unit (MCU), as using external memories would increase cost and energy usage. The data memory range between 2 and 32 kB, where 4 kB is the most typical choice[10]. As part of the data memory must be reserved for processing the sensed data and maintaining route and neighbor information, the remaining buffer space is low.

The amount of packets that fit to the buffer can be derived from the average packet size. As WSN traffic consists of sensor measurements or simple commands to actuators, a typical packet size is 32-64 B. For example, in IEEE 802.15.4 MAC maximum packet size is 128 B. Assuming 64 B packet size and 2 kB available buffer space, a buffer can hold 32 packets. As the packet must be buffered until it has been successfully routed, link errors combined with the burst nature of the WSN traffic increase buffer overflow probability significantly.

### 2.4. Methods for Reliability

Unreliability is compensated in the physical layer with channel encoding schemes that reduce packet errors. As channel encoding alone cannot guarantee reliability, network protocols utilize retransmissions. However, retransmissions only in Medium Access Control (MAC) layer are not sufficient, as packet errors and mobility cause link failures, thus causing packet drops.

The traditional networks use end-to-end transport protocols, such as Transmission Control Protocol (TCP), to guarantee reliability. Those protocols buffer a packet in the source node until it is acknowledged by the destination node. Using an end-to-end transport protocol in multihop WSNs has two drawbacks. First, a packet must be retransmitted again from the source node over several hops even if the previous transmission fails near the destination node. Thus, the communication overhead and energy consumption increase [11]. Second, WSNs often use reduced functionality devices that are dedicated to a specific task and have a very limited memory as source nodes. Therefore, source node buffering will quickly use all available buffers.

Multipath routing increases reliability by forwarding data via several paths. Then, a packet can be delivered even if routing on a certain path fails. The drawback of the multipath routing is increased messaging that consumes more energy and reduces network capacity. Uncontrolled multipath routing (e.g. flooding) would even have a negative impact on performance as the network congests and packets get dropped due to full buffers.

### 2.5. Related Research

The related research mainly aims at protocols that ensure reliable data delivery either via multipath routing [12],[13] or with a transport protocol that is suitable for WSNs [14–17]. These proposals introduce protocols that adapt to the network conditions, but the actual causes for the unreliability are not addressed in the literature.

Due to the data centric nature of the WSNs, few papers define the reliability as a probability that data is sensed in every region of the monitored area with a required minimum accuracy. In [18], reliability is defined based on estimated data generation intervals and the probability of a node failure. A model that analyzes reliability when several nearby nodes fail simultaneously due to the same reason is presented in [19]. The effect of the number of active reporting nodes to the reliability is modeled in [20]. The paper finds an optimal number of reporting nodes noting that too many nodes increase traffic and thus cause collisions and data loss, while too few nodes do not provide enough information.

An empirical study on reliability in outdoor and indoor WSNs is presented in [6]. The study finds that a network commonly has unequal link error rates and packet loss rates worse than 50% are common with heavy traffic. The results are based on Berkeley motes and TinyOS MAC that uses Carrier Sense Multiple Access/Collision Avoidance (CSMA/CA) protocol. The authors note that a CSMA protocol with virtual carrier sensing, such as S-MAC [2], can eliminate hidden node problems and thus have better performance. While the study presents practical behavior in WSNs, it does not consider causes or offer remedies to the unreliability.

Buffer space requirements when disseminating data from a single source to multiple nodes are studied in [21]. The paper proposes a buffer management algorithm that aims to reduce buffer requirements by buffering a packet only in a subset of all nodes. While the resulting delivery latencies and reliability is studied, the paper does not consider the effect of link errors. A queuing model for mesh networks utilizing sleeping is presented in [22]. The model considers the trade-off between wakeup probability, traffic arrival probability, queue size, and latency. In [23], end-to-end latency and reliability when transmitting a batch of packets is modeled. The effect of different single hop retransmission policies and PER is analyzed.

Shin *et al.* [24] study acknowledgments, buffering, bit error rate, and encoding schemes in WSNs. Reliability is evaluated using a CSMA-based MAC with flooding, Ad hoc On demand Distance Vector (AODV), and geographic routing protocols. The study suggests using hop-by-hop acknowledgments, message buffering, error correction scheme, and adjusting radio transmission range based on network density to reduce interference. While the study considers link errors, it does not evaluate the end-to-end latency caused by the different schemes.

## 3. ZigBee and TUTWSN Technologies

WSNs use two main approaches for medium access, TDMA and CSMA. In TDMA nodes communicate in non-overlapping time slots, whereas CSMA uses competition based channel access. TDMA has higher power saving potential as idle listening is minimized. However, TDMA is also more complex than CSMA as control signaling is required to distribute the time slots. The benefit of the CSMA is its flexibility as bandwidth is assigned on-demand basis. The drawback of the CSMA is that collisions that may be common when several source nodes compete over the wireless medium. In this paper, we evaluate reliability with ZigBee and TUTWSN protocols that represent the two medium access techniques.

### 3.1. ZigBee

**Figure 5.**
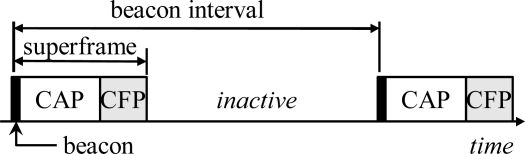
Superframe structure in IEEE 802.15.4 LR-WPAN with beacon enabled mode.

IEEE 802.15.4 Low Rate Wireless Personal Area Network (LR-WPAN) [25] defines physical and MAC layers for WSNs. LR-WPAN provides a synchronized low duty-cycle operation by optional beaconing mode, cluster-tree network topology, and a superframe structure used during the active period. A superframe consists of a beacon, Contention Access Period (CAP), and Contention Free Period (CFP) as shown in Figure 5. During the CAP, a coordinator receives data from the associated nodes using a slotted variation of CSMA. Normally, a node uses CAP but it can request for a dedicated time slot in CFP e.g. for ensuring low-latency or certain bandwidth. However, CFP can be used only for direct communication with the coordinator.

During the channel access of the IEEE 802.15.4, nodes avoid collisions by postponing their transmission by a random backoff period. After the backoff period, a node performs a Clear Channel Assessment (CCA) procedure to determine if the medium is free. A drawback in IEEE 802.15.4 is the absence of a mechanism to prevent hidden node problem, such as Request To Send/Clear To Send (RTS/CTS) procedure. While this simplifies the protocol, the performance degrades due to collisions [26].

ZigBee specifies application framework, device profiles, network layer, and security services for IEEE 802.15.4 LR-WPAN. The routing in ZigBee uses source initiated and routing table driven approaches. A source node generates a route request that is flooded to the target node, which replies with a route response message. The intermediate nodes update their routing tables so that they can forward data between source and target nodes. Only the routing table entry to the next hop neighbor that has the lowest routing cost is maintained. The cost is determined based on link reliability. Unreliable link is assigned with a high cost. Optionally, ZigBee allows reduced functionality devices without routing capacity. These devices route only along the cluster tree topology of IEEE 802.15.4. As beacon indicates the distance to the PAN coordinator that operates as the root of the cluster tree, devices can select a next hop among the neighbors that are nearest to the coordinator. Thus, route messaging is not required.

### 3.2. TUTWSN

TUTWSN consists of network protocols, sensor applications, hardware platforms, and tools and services for accessing sensor data from connected external networks [27, 28]. In this section, only MAC and routing layers are presented. Also, the differences between TUTWSN and ZigBee relevant to this research are discussed.

In TUTWSN, each cluster operates on its own frequency referred to as a cluster channel. A cluster head advertises its presence by sending network beacons using a common network channel. This way, instead of scanning through every possible cluster channel, a node can detect clusters rapidly by listening to the network channel.

**Figure 6.**
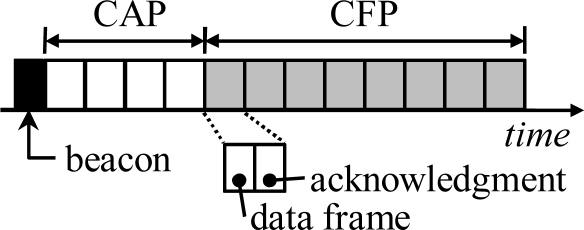
Superframe structure in TUTWSN MAC. CAP and CFP are divided into fixed time slots comprising two subslots, one for data frame and another for acknowledgment.

The superframe structure is similar to the IEEE 802.15.4. However, instead of using carrier sensing, CAP and CFP are divided into fixed time-slots as shown in Figure 6. Each time slot is further divide into two subslots, first subslot is for data frame and the following subslot is for acknowledgment. The use of contention free slots is preferred as it eliminates collisions and thus increases reliability [29]. The ALOHA based CAP is used only for joining a cluster and requesting reservations on CFP.

The use of contention free slots makes TUTWSN energy-efficient as contention is minimized. However, it also potentially increases forwarding delays on the presence of link errors, as a node must probably wait to the next active period for new transmission attempt. In CSMA-based protocols, like ZigBee, a node may try a new transmission immediately after a failed attempt.

In this paper, we use the following simple scheme for slot reservation. Each cluster member is always assigned with one slot per active period. If a node has queued frames, it asks for an additional slot by setting a flag in its data transmission. The cluster head assigns a slot during the ongoing access cycle and indicates the slot in the acknowledgment frame. The benefit of the scheme is that it divides bandwidth flexibly among the members according to the actual traffic requirements. Thus, the results obtained with the TUTWSN are near optimal when considering the practical implementations of TDMA-based slot assignment.

TUTWSN utilizes cost-based routing [30] that is commonly used in WSNs due to its simplicity and suitability to data centric networks. In the cost-based routing, each node is assigned with a cost that is calculated based on the distance to the sink. Thus, by forwarding data toward a neighbor that has a lower cost, a packet eventually reaches the sink. The routing exploits the notion that the traffic in a typical measurement WSN is highly asymmetric, as most of the traffic is routed from a node to a sink. For example, in a measurement network sink collects sensor values and may act as a gateway to Internet.

In TUTWSN, node initially searches its neighbors with a network scan. When a new neighbor is found, a node sends a cost request to it. A node selects the next hop and sets its cost based on the received replies. Additionally, nodes periodically recalculate the cost and broadcast an advertisement to their neighbors. This way, nodes can react to the changes in the network conditions as cost changes.

## 4. Simulation Results

In the simulations, we determine end-to-end reliability, latency, and availability in a typical low duty cycle WSN. As the synchronization affects the performance, we first study the effect of beacon losses. Then, we simulate the effect of network load, packet errors, and buffer sizes to the performance. For generalizable results, the simulations are performed both with ZigBee and TUTWSN protocols.

### 4.1. Simulation Scenario

Simulations were performed with Network Simulator 2 (NS2) version 2.31. As NS2 does not include ZigBee routing by default, the routing was implemented and the supplied IEEE 802.15.4 implementation was modified to support beacon scheduling as described in the ZigBee standard. The transceiver was configured to 2.4 GHz frequency band and 250 kb/s data rate. In ZigBee, IEEE 802.15.4 LR-WPAN used slotted and beacon enabled mode, because it is most suitable for low energy WSNs. Only one cluster channel was utilized, which results to a network scan time of one access cycle. Battery life extension was not used.

For comparable results, ZigBee and TUTWSN were configured with similar throughput and access cycle length. All simulations used 18 B application payload. In ZigBee, the beacon interval was 3.94 s and superframe length was 0.24 s. TUTWSN MAC used 4 s access cycle, 4 contention-based slots, and 8 reserved slots per access cycle. Time schedules ensuring that the active periods (superframes) of nodes within interference range do not overlap were used for both MACs.

The routing table driven approach in the ZigBee requires end-to-end messaging and can therefore be inefficient in a multihop network. However, as using the routing table is optional in the standard, the cluster tree routing was used instead. As a result, operation in both protocols is similar. A node finds a route locally by first performing a network scan and then selecting and associating to the neighbor that is nearest to the sink. Both TUTWSN and ZigBee routing used equal cost on each link, thus resulting into minimum hop forwarding.

**Figure 7.**
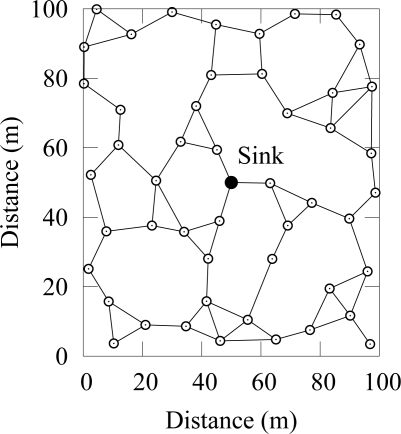
Simulation topology. A link is drawn between nodes within communication range.

Simulation scenario consisted of 50 randomly deployed nodes as shown in Figure 7. Two-ray ground propagation model was used with a maximum communication range of 20 m.

Nodes that acted as traffic sources generated packets to a sink node according to poisson distribution as it causes slight variation in traffic and thus gives more realistic results than sending traffic with constant intervals. The rate parameter λ for the poisson traffic was derived from the desired offered load *o* and the application payload as $\lambda =\frac{18B}{o}$.

The duration of each simulation was 3 hours. The results were averaged over 10 repetitions with randomly selected source nodes. To avoid path length from causing different results between repetitions, the source nodes had the same average hop count (4 hops) to the sink. In all results, 95% confidence interval was less than 10% of the measured values. Both end-to-end reliability and goodput were measured with a goodput metric that is the ratio of packets generated at the source node against the number of unique packets received at the destination node.

Packet errors were uniformly generated. Thus, each packet has the same probability of being discarded regardless of the packet length. The default seed in ns-2 was used for the random number generator. Unless otherwise stated, the buffer size is set to 500, which is high enough to guarantee that buffer drops do not occur due to momentary network errors.

### 4.2. Beacon Losses and Link Errors

**Figure 8.**
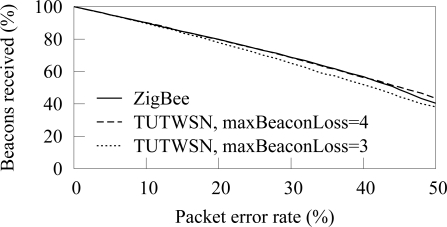
Beacon losses due to packet errors. TUTWSN is presented with synchronization loss after 3 and 4 consecutive beacon misses. ZigBee uses the default 4.

The amount of beacons received from a next hop neighbor is shown in Figure 8. Only the beacons that are received when a node is associated to the next hop neighbor are considered, as a node can forward its data only after the association. Thus, the result indicates forwarding capacity of a node. TUTWSN is presented with 3 and 4 as the maximum number of successive beacon misses before a synchronization is considered lost. In ZigBee, the default value of 4 beacon misses was used.

At low PERs, beacons are missed linearly according to the error rate. However, a high packet error rate increases the probability of missing several successive beacons, therefore causing a synchronization loss. On ZigBee, the synchronization is lost 2 and 8 times per hour on 30% and 40% PERs, respectively. On TUTWSN, the synchronization losses per hour are 5 and 12. After a synchronization loss, a node performs network scan and association procedures during which a node may not forward its data.

Allowing more beacon misses before assuming a link break is beneficial when synchronization losses are caused only by link errors. However, in a practical network, beacon losses might be caused by malfunctioned neighbor node or node mobility. Therefore, it may not be feasible to increase catch attempts significantly.

**Figure 9.**
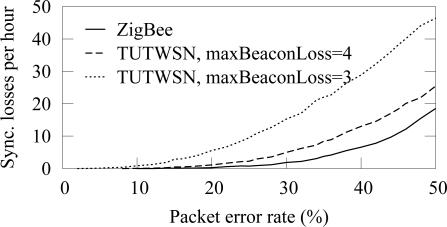
Synchronization losses due to several consecutive beacon misses.

The frequency of synchronization losses is presented in Figure 9. At low PERs, synchronization losses are rare. However, when the PER is 20% or more, synchronization fails often.

ZigBee and TUTWSN have similar behavior when the same number of beacon catch attempts is used. Still, on very high packet error rates (more than 40%) ZigBee receives less beacons than TUTWSN although it experiences slightly less synchronization losses. The behavior can be attributed to the complex association messaging of IEEE 802.15.4. During the association, a device (node) sends first an association message to a coordinator (cluster head). Then, the device requests for an association response with a data request. An association fails, if the transmission of any request or response fails. In comparison, TUTWSN proposes only one association request message, which is replied with an association response.

### 4.3. Hidden Node Problem in IEEE 802.15.4

In this simulation case, we examine the hidden node problem with IEEE 802.15.4 in low duty cycle multihop networks. As the hidden node problem occurs due to contention based channel access, reservation based protocols, such as TUTWSN, do not suffer from it.

**Figure 10.**
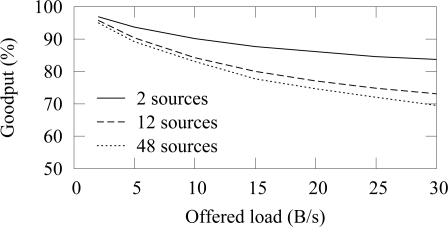
Goodput with different number of sources in ZigBee.

Figure 10 shows the goodput with different number of traffic sources. The offered load in the figure is the aggregate offered traffic from all nodes. Thus, the traffic generation interval on a source node is adjusted proportionally to the total number of source nodes, therefore resulting into a comparable aggregate offered load on each case. When the offered load is small, the goodput difference between different amount of source nodes is negligible. As the offered load is increased, the use of several source nodes decreases goodput significantly. Congestion is not an issue, because the network capacity exceeds the offered loads. It is possible that two nodes randomize the same backoff slot, which causes a collision despite of the CCA procedure but such event is rare and is recovered with a retransmission. Thus, the unreliability is caused by the collisions due to the hidden node problem. As more traffic enters the network, the collision probability increases and goodput decreases.

The low duty cycle operation increases the effect of the hidden node problem. Without the low duty cycle operation, a node can forward its data immediately which decreases the probability that two nodes transmit at the same time. However, in a low duty cycle network, a node must buffer data received during an idle period and wait until the active period of its next hop neighbor. Consequently, it is likely that several nodes try to transmit data at the beginning of an active period and a collision occurs between hidden nodes.

**Figure 11.**
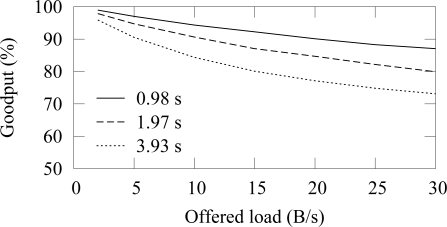
Goodput with different access cycle lengths in ZigBee. Duty cycle and throughput are the same on each case.

The effect of the access cycle length (beacon interval) to the reliability with 12 sources is shown in Figure 11. The duty cycle and thus the throughput are similar on each case. Because long access cycle length increases the probability that node has data to send during the next active period, the collision probability also increases.

To evaluate the reason why the goodput drops significantly, lets consider the probability that two transmissions beginning at the same time collide. The length of the random backoff period is 0..2*^BE^* − 1 backoff slots, where *BE* is a backoff exponent. *BE* ranges between *macMinBE* and *macMacBE* that have default values of 3 and 5, respectively. At the beginning of the channel access procedure, *BE* is initialized to *macMinBE*. If CCA fails, *BE* is increased up to *macMaxBE*. However, in the hidden node problem, CCA succeeds because the nodes do not hear each other, and therefore *BE* is equal to *macMinBE*. The transmission time of a data frame with 18 B payload and an acknowledgment frame equals to 6 backoff slots. As the default number of backoff slots is 2*^macMinBE^* = 8, there is 75% chance that two hidden node transmissions collide. Since the following retransmission attempts occur also at the same time, the probability that a transmission succeeds is very low.

The probability that transmission succeeds in *i*th backoff slot when another node has started channel access procedure at the same time (e.g. at the beginning of the active period) can be calculated as

(2) $$p_{i}=\{\begin{array}{ll} \frac{N-s-i}{N} & i<s\\ \frac{N-2\cdot s+1}{N} & s\leq i\leq N-s\\ \frac{i-s}{N} & N-s<i<N \end{array},$$

where *N* = 2*^BE^* is the total number of backoff slots and *s* is the data transmission time in backoff slots. The total transmission success probability is defined as

(3) $$P\{\text{tx succeeds}\}=\frac{1}{N}\cdot \sum_{i=0}^{N-1}p_{i}.$$

Few papers have proposed methods to eliminate the hidden node problem [31, 32]. The problem with these proposals is that they require modifications to the standard. We suggest two simple but efficient methods that minimize the effect of the hidden node problem. While these methods do not completely prevent collisions, the collision probability decreases which allows recovery via retransmission procedure.

The first method is to decrease the beacon interval. This causes a trade-off between network lifetime and reliability, because energy usage increases with shorter interval as beacons must be received more often [33].

The second method is to increase the value of the minimum backoff exponent. A large exponent decreases throughput but increases energy-efficiency as collisions are reduced. The trade-offs with the backoff exponent are studied [34], although the hidden node problem is not considered. In the remaining simulations, we set the minimum backoff exponent to 5 which decreases the collision probability between two hidden nodes from 75% to 32%, thus increasing the success probability of a frame transmission with retries from 68% to 99%.

### 4.4. Network Load and Packet Errors

Usually, only a part of the nodes are active in WSNs, e.g. nodes that detect an event, while the other nodes route data. To reflect this behavior, the network load was tested with 12 source nodes, which is 25% of the total node count.

**Figure 12.**
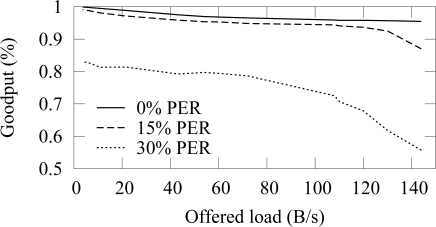
Goodput in ZigBee with varying offered network load and different packet error rates.

**Figure 13.**
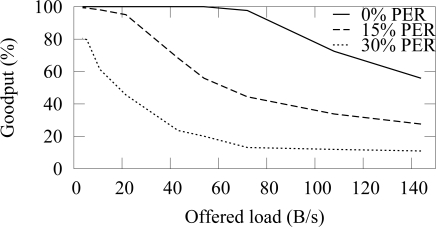
Goodput in TUTWSN with varying offered network load and different packet error rates.

Figure 12 and Figure 13 shows the end-to-end reliability with different offered loads and packet error rates in ZigBee and TUTWSN, respectively. Because either protocol does not include a mechanism to ensure end-to-end reliability, the goodput is below 100% when packet errors occur. In ZigBee, the hidden node problem decreases the goodput slightly.

The difference between the goodput of 0%–15% PERs is small with both protocols. With the 30% PER, goodput decreases drastically. This is consistent with the earlier observation that the synchronization losses increase significantly at 20%–30% PERs, thus causing packet drops.

When the offered load exceeds the network capacity, the network congests and the packets are dropped due to full buffers. This happens after 140 B/s load with ZigBee and after 60 B/s load with TUTWSN. However, with the used configuration, both protocols have the theoretical capacity of 2 pkt/s when only one node is utilizing the medium. In ZigBee, this value was calculated based on the average backoff times (BE=5) and data, acknowledgment, and interframe times. TUTWSN has worse performance because of the inflexibility of the TDMA-based channel access. Each member node is assigned with one slot per access cycle even if they have nothing to send, which wastes capacity. Additionally, the CSMA-based channel access in ZigBee benefits when several nodes compete over the medium.

Increasing the number of competing nodes in ZigBee has two effects. First, the collision probability increases as two nodes may select the same backoff time. In the simulated topology, nodes have 1–4 neighbors. Thus, only a few neighbors compete over the medium, which keeps the collision probability small. Also, as the backoff exponent was increased, the hidden node problem that would otherwise decrease the throughput is not significant. Second, since several nodes independently randomize their backoff times, the average backoff time between transmissions is reduced proportionally to the number of the competing nodes. That is, idle times are reduced which increases the utilization of the medium and thus the throughput.

When packet errors occur, capacity is lost due to missed beacons and retransmissions which causes the network to congest earlier. TUTWSN suffers from high PERs, because the simple channel reservation scheme used in the simulations fails in requesting additional reservations due to packet errors. As a result, a node must wait for a reserved slot until the next active period, which wastes capacity.

**Figure 14.**
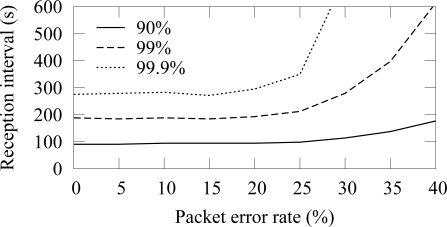
Availability intervals with 90%–99.9% availability in ZigBee (12 sources, 5.5 B/s offered load).

**Figure 15.**
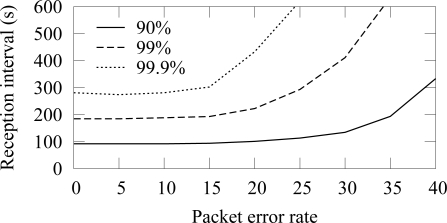
Availability intervals with 90%–99.9% availability in TUTWSN (12 sources, 5.5 B/s offered load).

The availability in ZigBee and TUTWSN with 12 source nodes and 40 s traffic generation interval (5.5 B/s aggregate load) is presented in Figure 14 and Figure 15, respectively. To express the effect of different PERs, the figure does not include all availability percentages as in Figure 4. Instead, the time intervals required reach the 90%, 99%, and 99.9% availabilities are presented.

In both protocols, 90% availability is obtained with 100 s interval, while 99% availability requires 200 s interval. The behavior is due to poisson generated traffic that causes variation between data generation intervals. Between 0%–15% PERs, the availability does not decrease notably, because MAC layer retransmissions are enough to compensate the packet errors and links break rarely. The reception interval that is required to reach 90% availability does not notably increase until the PER is higher than 25%. For 99% and 99.9% availability, the corresponding PER thresholds are 25% and 15%. Clearly, the traditional 3 retransmission attempts typically used in MAC protocols are not enough to provide sufficient availability when the PER is larger than 15%–20%.

### 4.5. Effect of Buffer Size

A packet buffer must be able to hold received packets until they can be forwarded. In low duty cycle networks, a node cannot forward packets immediately, but must be able to hold packets received during own active period until the active period of the next hop occurs. Assuming that a cluster head is routing data generated by *n* nodes, the average buffer requirements *B* can be calculated as

(4) $$B=n\cdot \frac{t}{i},$$

where *t* is the buffering time and *i* is the traffic generation interval. When packet errors do not occur, the buffering time *t* equals to the access cycle length. Packet errors cause beacon misses and thus increase the buffering time.

**Figure 16.**
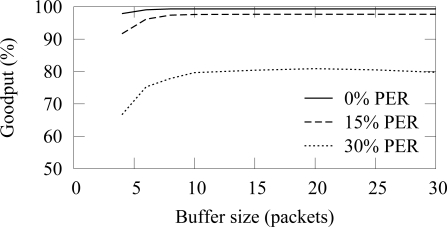
End-to-end reliability in ZigBee with different buffer sizes (12 sources, 15 B/s offered load).

**Figure 17.**
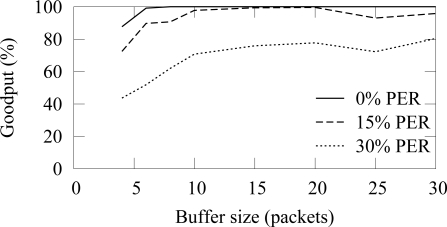
End-to-end reliability in TUTWSN with different buffer sizes (12 sources, 15 B/s offered load).

In the buffer simulation, 12 nodes generated packets with 15 s average interval (15 B/s offered load). Thus, the average buffer usage should be 3.2 with the used 4 s access cycle. The end-to-end reliability with the ZigBee and TUTWSN is presented in Figure 16 and Figure 17, respectively. Without packet errors, 100% the maximum goodput is reached with the buffer size of 6 as expected. When packet errors occur, larger buffer sizes allow retransmissions and error recovery. TUTWSN requires more buffers than ZigBee because its routing protocol transmits periodic route advertisements to neighbors, thus causing an additional buffer requirement of 1–2 packets. The ZigBee used the simple cluster tree routing algorithm that does not cause route messaging but is only able to route to the PAN coordinator.

## 5. Reliable Data Forwarding Algorithm

In a WSN that is deployed for control and monitoring purposes the reliability of a single packet is very important as critical alert messages may not be missed. Because using a protocol that requires buffering packets on a source node such as traditional TCP is not desirable, a distributed end-to-end scheme for WSNs is required to ensure reliability and availability.

Based on the results on the importance of buffer space control, we propose a simple data forwarding algorithm. While there are other reliability schemes proposed for WSNs, such as WSN transport protocols [14–17] and distributed TCP caching [35, 36], the benefit of the presented scheme is its simplicity and applicability to common wireless MAC and routing protocols. This algorithm does not make assumptions on the used routing protocols and does not introduce communication overhead.

The reliable data forwarding algorithm relies on the co-operation and cross-layer design between MAC and routing layers. The algorithm uses anycast type forwarding and is an extension to the well known automatic repeat request scheme in which the number of repeats is not limited (ARQ-∞ scheme). Therefore, the algorithm can be utilized in any WSN that uses retransmissions. We extend the ARQ-∞ scheme by allowing recovery from a broken link and utilizing alternate links. This way, a packet is reliably forwarded to the target node unless a forwarding node fails completely, e.g. runs out of energy. This simple scheme is feasible, as link failures can be assumed to be much more common than node failures.

**Figure 18.**
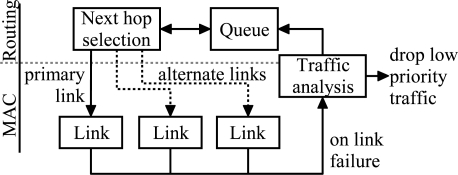
Routing layer end-to-end reliability scheme with intermediate node buffering.

The algorithm is presented in Figure 18. A packet is passed to the MAC layer after the next hop selection. If the transmission fails due to link failure or because the maximum number of transmission attempts was reached, the packet is passed back to the routing layer and queued until a valid route is found. However, as the sensed values in a typical measurement network are highly redundant, it is sometimes more energy-efficient and therefore desirable to drop low priority packets than try to send them indefinitely. To allow this, the data forwarding scheme analyzes the type of the packet after the link failure and drops a low priority packet.

To allow fast recovery from link errors, alternative links to the sink are maintained. If the transmission on the primary link fails, one of the alternative links can be used without the time consuming network scan. This way, a packet can be routed via an alternative link if the primary link is experiencing momentary interference or high load. Reactiveness in such situations can be increased by decreasing the number of transmission attempts per link e.g. by performing only one retransmission attempt. Thus, interference is detected and an alternative link is used earlier.

### 5.1. Feasibility Analysis

To analyze the feasibility of the ARQ-∞ based scheme, energy-efficiency is evaluated with the expected number of transmissions required to reach a destination node. Other aspects of performance, such as latency, are not evaluated. The transmission count is compared against other common reliability schemes: multipath routing and traditional transport protocol. To simplify the evaluation, the analysis assumes static network topology and assumes that MAC layer acknowledgment for a data frame is always received.

An analysis of transport protocols that utilize distributed caching is omitted as their performance depend on how well the data cache is distributed. In the worst case, data is cached near source node and the number of transmitted attempts is similar to the traditional transport protocol. In the best case, data is cached at the hop in which the transmission failed and the number of transmission attempts is similar to the ARQ-∞ scheme.

In the ARQ-∞ scheme, the expected number of transmission attempts on a path consisting of *L* hops is

(5) $$T_{\mathrm{ARQ}-\infty }=\sum_{i=0}^{L-1}a_{i}^{M},$$

where $a_{i}^{M}$ is the number of transmissions on *i*th hop. It is calculated based on the link reliability *r_i_* as

(6) $$a_{i}^{M}=1+\sum_{j=1}^{M}(j+1)\cdot {\left(1-r_{i}\right)}^{j}\cdot r_{i},$$

where *M* is the limit for transmission attempts. For example, in IEEE 802.15.4 and IEEE 802.11, the default number of transmission attempts is 4 (3 retries). In ARQ-∞ scheme the number of attempts is not limited (*M* = ∞) and (6) can be rewritten as

(7) $$a_{i}^{\infty }=1-r_{i}+\frac{1}{r_{i}}.$$

In multipath routing utilizing *n* alternative paths the expected number of transmission attempts is

(8) $$T_{\mathrm{mp}}=\sum_{j=0}^{n-1}\sum_{i=0}^{L_{j-1}}a_{i}^{M},$$

where *L_j_* is the path length of the *j*th path. The equation assumes that multiple paths do not converge, thus representing the simplest case of controlled multipath routing.

The analysis with the transport protocol is slightly more complex as a packet drop on any link on the route causes retransmission at the source node. This analysis assumes that retransmission timers are optimal and do not trigger while packet is still being transmitted. Also, we do not consider batch transmissions, which would decrease overhead as several packets can be acknowledged with one reply packet.

The expected number of transmissions on each hop depends on the position of the hop at the route. If transmission succeeds on a certain hop but fails at later hop, the packet must be retransmitted again. Therefore, we calculate the probability that the transmission succeeds in hops *i..L −* 1 as

(9) $$D_{i}=\prod_{j=i}^{L-1}1-{\left(1-r_{j}\right)}^{M},$$

where *r_j_* the packet error rate at *j*th link and *M* is the transmission limit. The total number of transmission attempts at *i*th hop is defined as

(10) $$A_{i}=a_{i}^{M}\cdot \left(1+\sum_{j=1}^{\infty }{\left(D_{i}\right)}^{j}\cdot (1-D_{i})\right)=a_{i}^{M}\cdot \left(D_{i}+\frac{1}{1-D_{i}}\right).$$

Finally, the expected number of transmissions to deliver packet from a source to the destination defined as

(11) $$T_{\mathrm{tp}}=\sum_{i=0}^{L-1}A_{i}.$$

In a transport protocol, the destination confirms the data packet with an acknowledgment. If the source does not receive the acknowledgment, a timer triggers and the packet is retransmitted. Thus, the total number of transmissions is modified with the expected number of times the acknowledgment needs to be send as

(12) $$T_{\mathrm{tpa}}=T_{\mathrm{tp}}\cdot \left(1+\sum_{i=1}^{\infty }(i+1)\cdot {\left(1-D_{0}\right)}^{j}\cdot D_{0}\right)=1-D_{0}+\frac{1}{D_{0}},$$

where *D*_0_ is the probability that an acknowledgment is successfully delivered over the whole path and is calculated with (9).

To analyze the performance of difference schemes, we consider a route consisting of 5 hops. In this scenario, the multipath routing utilizes 2 alternative paths with at most 3 retransmission attempts per link. Both paths are equally long and have similar error rates. To evaluate the effect of retransmission attempts to the amount of messages, transport protocol is examined with 2 retransmission attempts in addition to the typical 3 attempts.

**Figure 19.**
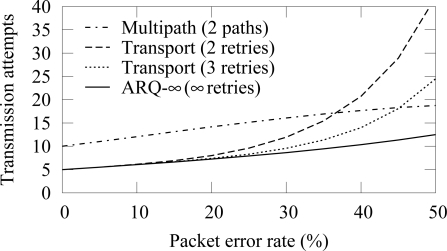
Complexity of different routing schemes with a route consisting of 5 hops having equal packet error rate.

The transmission count when all links have equal packet error rates is shown in Figure 19. With low error rates, ARQ-∞ scheme and transport protocol perform equally. As the PER increases, the message count in the transport protocol increases rapidly because packet drop in any part of the route causes retransmission from the beginning of the route. The smaller amount of retries increases the drop probability and thus the total message count. The transmission count with the multipath routing is always high due to the message delivery via several routes.

**Figure 20.**
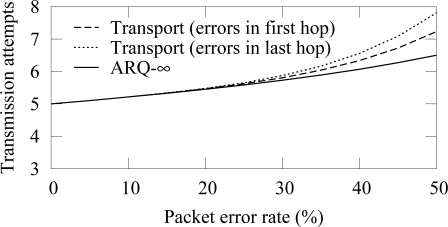
Complexity of different routing schemes with a route consisting of 5 hops. Only one hop is experiencing packet errors.

The message count when only one hop is unreliable is shown in Figure 20. Multipath routing is not evaluated, as its message count would be highest with all examined PERs. The transport protocol is evaluated separately when the first and the last hop in the routing path from a source to a destination are erroneous, showing that more transmissions are required when the erroneous link is near the destination. Both cases use 3 retransmissions attempts. Again, ARQ-∞ scheme performs better than the transport protocol.

As a conclusion, ARQ-∞ based reliable routing algorithm is an efficient method to ensure reliability in a static network. The drawback of the generic ARQ-∞ scheme is that packet is dropped on a link break or when route to the destination is lost. The reliable routing algorithm corrects this drawback by rerouting the packet locally instead of dropping it.

It should be noted, that although the analysis indicates that the multipath routing has the worst energy usage, it has other benefits. As a packet is routed via several paths, the end-to-end latency can be small even when one route experiences problems. Still, unlike a transport protocol and ARQ-∞, the multipath routing by itself does not guarantee reliability.

### 5.2. Simulation Results

The reliable data forwarding algorithm was simulated with ZigBee and TUTWSN. ZigBee uses only the primary link, as including alternative links would have required significant modifications to the standard. TUTWSN maintains one alternative link.

**Figure 21.**
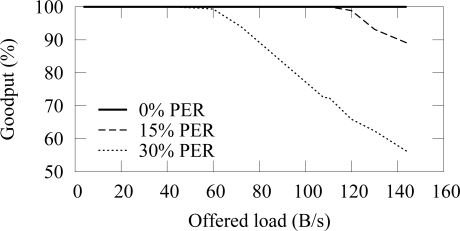
The effect of network load to the goodput in ZigBee using the reliable data forwarding.

**Figure 22.**
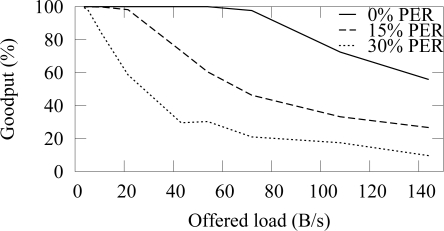
The effect of network load to the goodput in TUTWSN using the reliable data forwarding.

Figure 21 and Figure 22 shows the end-to-end reliability with different offered loads in ZigBee and TUTWSN, respectively. As TUTWSN do not suffer from hidden nodes, the results are identical to the case that did not use the algorithm when the PER is 0%. When packet errors occur, the scheme is able to deliver a packet reliably until the network congests and packets are dropped due to full buffers.

**Figure 23.**
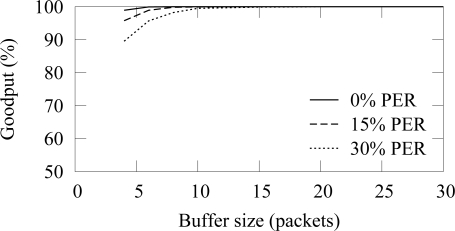
The effect of buffer size to the reliability in ZigBee using the reliable data forwarding algorithm (12 sources, 15 B/s offered load).

**Figure 24.**
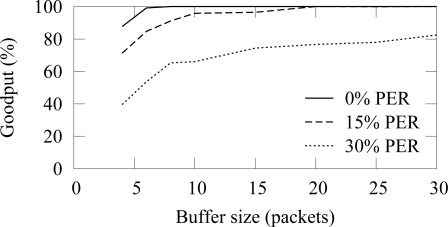
The effect of buffer size to the reliability in TUTWSN using the intermediate buffering scheme algorithm (12 sources, 15 B/s offered load).

The effect of practical buffer size limitations with 15 s packet generation interval (15 B/s aggregate load) is presented in Figure 23 and Figure 24. The reliable forwarding algorithm has better reliability with the same buffer space than the cases that did not use the algorithm.

**Figure 25.**
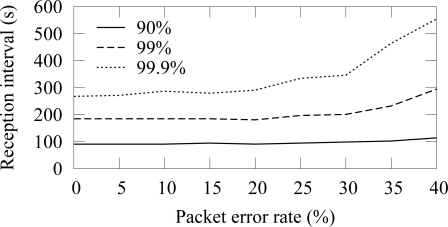
Availability in ZigBee using the reliable data forwarding (12 sources, 5.5 B/s offered load).

**Figure 26.**
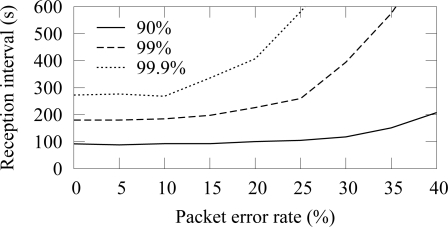
Availability in TUTWSN using the reliable data forwarding (12 sources, 5.5 B/s offered load).

The availability with 12 source nodes and 40 s packet generation interval (5.5 B/s aggregate load) is presented in Figure 25 and Figure 26. With the reliable forwarding, the interval that is required to reach a certain availability is significantly smaller at higher PER values. In ZigBee, the interval for 99.9% availability does not increase drastically until after 30% PER. The similar PER threshold without the reliable data forwarding was 20%.

**Figure 27.**
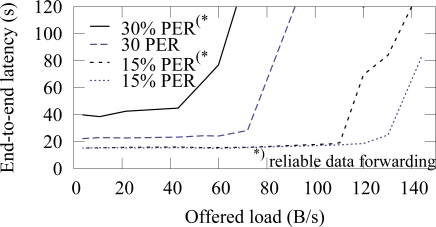
Average end-to-end latency with different packet error rates in ZigBee with and without the reliable data forwarding algorithm.

The end-to-end latencies with and without the reliable data forwarding algorithm in ZigBee are presented in Figure 27. The algorithm does not increase latency, when the PER is 15%. On the higher error rate, latency increases due to retransmissions. Without the algorithm, network congests after 70 B/s offered load (latency rises rapidly in the figure) at 30% PER. With the algorithm, the congestion occurs earlier at 50 B/s, thus indicating that the algorithm causes 30% overhead. ¿From the results we conclude that the algorithm is feasible for all purposes up to 30% PER. On a higher PER, the overhead and latencies increase significantly and the suitability of the algorithm depends on the application scenario. That is, the algorithm is still usable for example on lightly loaded measurement networks.

According to simulations, the reliable data forwarding algorithm guarantees reliability when the network traffic is sufficiently low. The drawback of the algorithm is that it slightly increases traffic due to retransmissions, which can lead to congestion. However, with the realistic PER values of 0%–30% the congestion occurs only slightly earlier than in the network without the algorithm. The effect of such congestion could be reduced by limiting the maximum buffering time for a packet on a node. Additionally, different buffering priorities would enable low latencies for high priority traffic.

## 6. Conclusions

This paper analyzes end-to-end QoS in synchronized low duty cycle networks. The contributions of this paper are summarized as follows

- We define two metrics to measure the WSN performance: reliability and availability. The reliability is the traditional metric that measures the probability of successful transmission. The availability measures the time that a node has been active, which makes it well suited for data centric WSNs.
- An analysis on the effect of synchronization with beacons in a low duty cycle network shows that significant amount of network capacity is lost due to missed beacons. The capacity loss is equal to the PER, when PER is between 0%–20%. On higher error rates, synchronization losses become common and the capacity loss increases. In a typical WSN MAC, the reduction is 31% and 44% for 30% and 40% PERs, respectively. The result motivates developing more robust beaconing schemes to increase the beacon reception probability, such as sending multiple successive beacons. In addition, to reduce the downtime after a synchronization failure, association procedure should be fast and robust e.g. by using only short message exchange.
- We analyze the hidden node problem in IEEE 802.15.4 contention-based channel access and note that the hidden nodes cause a significant reliability problem in practical low duty cycle networks. Even with few competing nodes, the end-to-end goodput drops by 20%. As a result, we propose adjustments to the backoff exponent and access cycle lengths that decrease the collision probability significantly.
- In our analysis on network load against packet errors, we found that the typical 3 retransmissions per link are enough to provide good availability when PER is less than 15%. With higher packet error rates, an end-to-end reliability scheme is required.
- We show that preparing buffer space only based on the expected traffic is not enough. Packet errors significantly increase the buffer requirements. In low duty cycle networks, sufficient buffer space is required to prevent data loss as a node cannot forward its data due to beacon misses and synchronization losses. In our simulations, compared to the network without packet errors, 30% PER doubled the buffer requirements.
- We present a simple but efficient reliable data forwarding algorithm suitable for resource constrained WSNs. The benefit of the reliable data forwarding algorithm is its ease of implementation and applicability to several existing MAC and routing protocols. The algorithm buffers a packet in intermediate nodes along a route until the packet is successfully forwarded. The buffering scheme guarantees end-to-end reliability, assuming that network errors are caused only by link failures (node failures do not occur).

This paper identifies and evaluates the factors affecting low duty cycle WSN performance. The following findings are a subject for further work. First, while the proposed methods to reduce the hidden node problem in ZigBee are shown as valid, analytic evaluation on the trade-offs and optimal parameters on different duty cycles, traffic loads, and packet error rates is required. Second, our ZigBee evaluation did not utilize the contention-free channel access, as its performance depends on the reservation policy that is not explicitly specified in the standard. The effect of different reservation methods to the performance should be evaluated. Last, the reliable data forwarding algorithm should be compared against other end-to-end reliability schemes, such as WSN transport protocols or TCP modifications for wireless multihop networks. Also, the impact of utilizing alternative links to the reliability and energy-efficiency should be examined more carefully.
